# Evaluation of Two Plasma pTau217 Simoa Assays Against Amyloid‐PET

**DOI:** 10.1002/alz.087493

**Published:** 2025-01-09

**Authors:** Daniel Figdore, Joshua A Bornhorst, Jonathan Graff‐Radford, Prashanthi Vemuri, David S. Knopman, Clifford R. Jack, Ronald C. Petersen, Alicia Algeciras‐Schimnich

**Affiliations:** ^1^ Mayo Clinic, Rochester, MN USA

## Abstract

**Background:**

Tau phosphorylated at threonine 217 (pTau217) is one of the most promising blood‐based biomarkers of Alzheimer’s disease (AD). This study compares the performance of two plasma pTau217 immunoassays on the Simoa platform for detection of abnormal amyloid‐PET.

**Method:**

Plasma samples from 112 individuals without cognitive impairment and 114 with mild cognitive impairment (MCI) or AD related dementia were evaluated. Testing was performed on the Quanterix Simoa® HD‐X Analyzer using the ALZpath Simoa® pTau217 and the Simoa® ACC pTau217 (Janssen antibodies) commercial assays. Amyloid‐PET imaging was performed using ^11^C‐Pittsburgh Compound B and a standard uptake value ratio (SUVR) cutoff of ≥1.52 (≥25 Centiloid) was used for abnormal (n= 147) vs. normal (n= 79) classification. Passing‐Bablok regression analysis and Spearman’s correlation coefficient (rho) were used to estimate bias and correlation between the assays, respectively. Correlation of each assay with Amyloid‐PET SUVR was evaluated. Amyloid‐PET status as the outcome and plasma pTau217 as predictor was used to estimate the area under the receiver operating characteristic (ROC) curve (AUC).

**Result:**

The pTau217 concentrations between the two assays showed an excellent correlation (Spearman’s rho= 0.94 [95% CI= 0.93 to 0.96, p<0.0001]). However, the Passing‐Bablok regression analysis equation for ALZpath (x‐axis) and ACC (y‐axis) was y= 0.1324x ‐ 0.0019, showing that measured pTau217 concentrations with the ALZpath assay were higher (∼87%) than with the ACC assay (concentrations ranged 0.057 to 3.5 pg/mL and 0.003 to 0.5 pg/mL, respectively). Correlations between amyloid‐PET SUVR and the two pTau217 immunoassays were almost identical with Spearman’s rho= 0.70 (95% CI= 0.62 to 0.76, p<0.0001) for ALZpath and 0.69 (95% CI= 0.61 to 0.75, p<0.0001) for ACC. The AUC for detection of an abnormal amyloid‐PET among the MCI/AD group was 0.88 (95% CI= 0.81 to 0.95) for ALZpath, and 0.89 (95% CI= 0.83 to 0.96) for ACC.

**Conclusion:**

Analytically, the two Simoa® pTau217 assays correlate well with each other, but results are not interchangeable due to a significant proportional bias. Clinically, the performance of the assays was largely equivalent for the detection of abnormal amyloid‐PET by AUC analysis based on their almost identical correlations with amyloid‐PET SUVR.

